# An Investigation of Active Sites for electrochemical CO_2_ Reduction Reactions: From In Situ Characterization to Rational Design

**DOI:** 10.1002/advs.202003579

**Published:** 2021-03-03

**Authors:** Yuqin Zou, Shuangyin Wang

**Affiliations:** ^1^ State Key Laboratory of Chem/Bio‐Sensing and Chemometrics Provincial Hunan Key Laboratory for Graphene Materials and Devices College of Chemistry and Chemical Engineering the National Supercomputer Centers in Changsha Hunan University Changsha 410082 P. R. China

**Keywords:** active site, carbon‐based electrocatalyst, electrochemical CO_2_ reduction, in situ characterization, metal‐based electrocatalyst, metal‐organic frameworks‐based electrocatalyst

## Abstract

The electrochemical carbon dioxide (CO_2_) reduction reaction (CO_2_RR) is among the most promising approaches used to transform greenhouse gas into useful fuels and chemicals. However, the reaction suffers from low selectivity, high overpotential, and low reaction rate. Active site identification in the CO_2_RR is vital for the understanding of the reaction mechanism and the rational development of new electrocatalysts with both high selectivity and stability. Herein, in situ characterization monitoring of active sites during the reaction is summarized and a general understanding of active sites on the various catalysts in the CO_2_RR, including metal‐based catalysts, carbon‐based catalysts, and metal‐organic frameworks‐based electrocatalysts is updated. For each type of electrocatalysts, the reaction pathway and real active sites are proposed based on in situ characterization techniques and theoretical calculations. Finally, the key limitations and challenges observed for the electrochemical fixation of CO_2_ is presented. It is expected that this review will provide new insights and directions into further scientific development and practical applicability of CO_2_ electroreduction.

## Graphical abstract

Electrochemical reduction of CO_2_ to useful fuels and chemicals is of fundamental significance for carbon recycling but remains a great challenge. A comprehensive review of the essential advances in electrochemical reduction of CO_2_ is provided. This review will provide new insights into the further in situ characterization development and active sites identification of CO_2_ electroreduction.

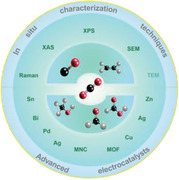

## 1 Introduction

Carbon dioxide (CO_2_) is constantly and continuously accumulating in the atmosphere due to the global consumption of fossil fuels, which leads to detrimental environmental pollution and climate change concerns. CO_2_ conversion to valuable chemicals is a possible solution to these issues.^[^ ^1^ ^]^ In recent years, the electrochemical CO_2_ reduction reaction (CO_2_RR) has attracted conspicuous attention.^[^ ^2^ ^]^ The process is controllable with electrode potentials and reaction temperature, renewable energy is used to power the process, and electrochemical reaction systems are compact, modular, on‐demand, and facile for scale‐up applications.^[^ ^3^ ^]^

However, the reaction rates and energy efficiencies of CO_2_RR are limited, even in the presence of electrocatalysts.^[^ ^4^ ^]^ Moreover, the selectivity of these catalysts remains low due to complicated reaction mechanisms.^[^ ^5^ ^]^ Even though CO_2_ is a stable molecule and the first electron transferred to form a CO_2_^−^ radical intermediate is carried out at a highly negative redox potential (−1.9 V vs. normal hydrogen electrode), stabilization of the CO_2_^−^ radical or other intermediates on the surface of the electrocatalyst is the first step of CO_2_RR.^[^ ^6^ ^]^ Meanwhile, the desorptive properties of intermediates on different electrocatalysts determine the obtained product. Thus, the selection of an appropriate electrocatalyst is critical to the reduction of CO_2_ at a low overpotential with high selectivity of the final, specific product.

Routes for CO_2_RR can be realized through multiple electron transfers in aqueous solution with suitable electrocatalysts. The main reduction products of the two‐, four‐, six‐, eight‐, and twelve‐electron reduction pathway are CO/HCOOH, formaldehyde, methanol, methane, ethylene/alcohols, respectively.^[^ ^7^ ^]^ Based on a thermodynamic study, a variety of half‐reactions and their corresponding electrode potentials versus reversible hydrogen electrode (RHE) in aqueous solution (pH = 7, at 25 °C, 1 atm, and 1.0 m concentration of other solutes) are listed in **Table** **1**.

**Table 1:** Representative half reactions and reduction potential of CO_2_‐reduction reactions in aqueous solutions

Half‐electrochemical reactions	Electrode potentials [V_RHE_] at pH = 7
CO_2_(g) + 2H^+^ + 2*e* ^−^ → HCOOH(1)	−0.250
CO_2_(g) + 2H^+^ + 2*e* ^−^ → CO(g) +H_2_O	−0.106
CO_2_(g) + 4H^+^ + 4*e* ^−^ → HCHO(1) + H_2_O(1)	−0.070
CO_2_(g) + 6H^+^ + 6*e* ^−^ → CH_3_OH(1) + H_2_O(1)	0.016
CO_2_(g) + 8H^+^ + 8*e* ^−^ → CH_4_(1) + 2H_2_O(1)	0.169
2CO_2_(g) + 12H^+^ + 12*e* ^−^ → C_2_H_4_(g) + 4H_2_O(1)	0.064
2CO_2_(g) + 12H^+^ + 12*e* ^−^ → C_2_H_5_OH(1) + 2H_2_O(1)	0.084

To obtain two‐electron transfer products, four reactions related to the activation of CO_2_ were considered:

(1) $$*+CO_{2}+H^{+}+e^{-}\to \mathrm{COOH}$$

(2) $$*+CO_{2}+H^{+}+e^{-}\to \mathrm{OCHO}$$

(3) $$*+CO_{2}+e^{-}\to \mathrm{CO}{}_{2}^{-}$$

(4) $$*+H^{+}+2e^{-}\to^{*}H^{-}$$

It is has been studied and now understood that *COOH is the most likely first intermediate for CO production, and *OCHO is the most likely intermediate for HCOOH formation.^[^ ^7^ ^]^ Experimental results agree with this theoretical prediction: post‐transition metals (Sn and Bi) prefer to bind CO_2_ via oxygen atoms and produce HCOOH as the main product, whereas transition metals (Ag, Au, and Zn) prefer to bind CO_2_ via carbon atoms.^[^ ^8^ ^]^ Over the past few decades, many electrocatalysts have been reported with improved reaction rates and high energy efficiency.^[^ ^1^ ^]^ Meanwhile, deeper knowledge and understanding of the reaction mechanism and pathway have been investigated using in situ techniques.

**Figure 1:**
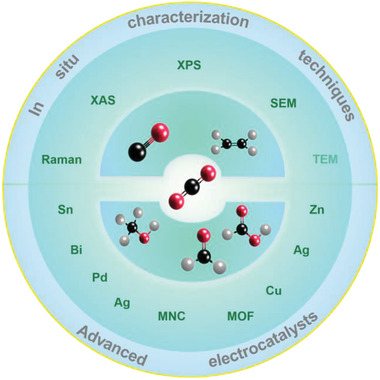
Overview of themes discussed in this review.

In this review, we will discuss the recent progress and future perspectives of heterogeneous electrochemical CO_2_RR (**Figure** **1**). The first part of the paper discusses the in situ techniques applied to investigate the active sites of CO_2_RR. Then, a summary of the various strategies utilized to achieve high selectivity via rational design of the catalyst is described, such as metal and metal‐based nanostructured compounds, carbon‐based catalysts, and metal‐organic frameworks (MOF)‐based electrocatalysts. For each type of electrocatalysts, the reaction pathway and real active sites are proposed based on in situ characterization techniques and theoretical calculations. Finally, we present observed challenges and limitations of the electrochemical fixation of CO_2_.

## 2 In Situ Characterization Techniques

Identifying active sites or species is a precondition for the rational design of catalysts exhibiting both high activity and high selectivity toward valuable products in CO_2_RR.^[^ ^9^ ^]^ In situ characterization techniques, which investigate the CO_2_RR process under real operating conditions and provide a deeper understanding of reaction mechanisms, material structures, and surface sites, are critical tools used to address these issues.

### 2.1 Raman Spectroscopy

Raman spectroscopy can detect inelastically scattered photons from a sample whose vibrational modes feature polarizability changes when a visible light laser from the monochromatic light is utilized. Due to the different rotational and vibrational states of various molecules, Raman spectroscopy is complementary to infrared spectroscopy (IR). More importantly, water has a low Raman scattering cross‐section, so an aqueous electrolyte can be used for in situ Raman spectroscopy.

**Figure 2:**
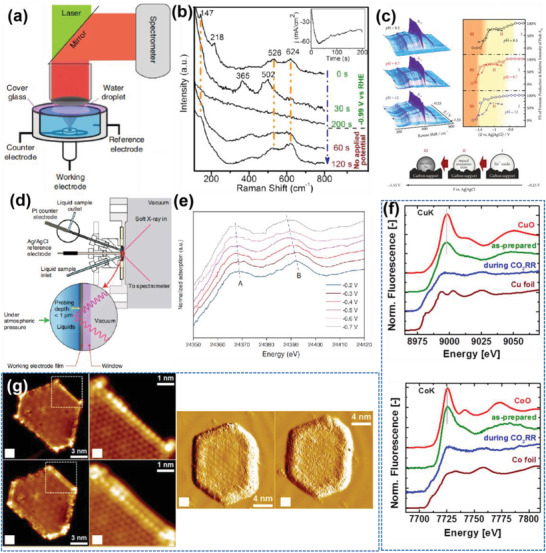
a) Schematics of operando Raman setup with water droplet in between the cover glass and lens to minimize the refractive index difference in the optical pathway; Reproduced with permission.^[^ ^9^ ^]^ Copyright 2015, Nature Publishing Group. b) In situ Raman spectra and corresponding chronoamperogram (inset) of 1.7 µm film at −0.99 V in 0.1 m KHCO_3_. Reproduced with permission.^[^ ^10^ ^]^ with permission from American Chemical Society. c) Left: The potential dependence of the Raman spectra for each studied pH. right: The relative intensities of the Sn_IV_‐related A_1g_ Raman peaks (○, solid line) and the FE of formate production (×, dashed line) as a function of electrode potential. In the three distinct potential regions represented by the shaded background, the catalyst is in the form of fully oxidized SnO_2_ (I), a partially reduced compound of mixed oxidation state (II) and completely reduced metallic Sn (III), as illustrated by the scheme of below part. Reproduced with permission.^[^ ^12^ ^]^ Copyright 2015, American Chemical Society. d) Electrochemical flow cell configuration used for operando X‐ray measurement. e) In situ XANES spectra of Pd K‐edge for Pd octahedra; f) top: Cu K‐edge, down: Co K‐edge XANES and respective EXAFS data of Cu_50_Co_50_ NPs of 11.6 nm measured as prepared in air and under operando CO_2_RR at −1.1 V_RHE_ in 0.1 m KHCO_3_. Reference spectra from bulk Cu, Co foils and CuO and CoO are also shown for comparison. Reproduced with permission.^[^ ^15^ ^]^ Copyright 2017, Royal Society of Chemistry. g) Interaction between CeO*_x_*/Au(111) and CO_2_. Reproduced with permission.^[^ ^30^ ^]^ Copyright 2018, Wiley‐VCH.

A reaction cell configuration is shown in **Figure** **2**a. The cell was designed to optimize light loss reduction as the incident and reflected laser pass through a thin (mm−cm) layer of the electrolyte with the aid of an optically transparent window. Reference and counter electrodes were positioned close to the working electrode to give accurate electrochemical signals and affixed in a position out of the light of sight of the laser beam. The preparation of working electrodes for in situ Raman measurements usually follows the same procedure as constructing electrodes for electrocatalytic experiments and requires no other specific treatments.

So far, the application of in situ Raman in CO_2_RR is confined to catalyst state monitoring during the electrocatalytic process. Real active sites are critical for the design of effective electrocatalysts. For metal oxides, researchers generally assume that the real activity site is the reduced metal surface, since the electrocatalytic reaction takes place at the cathodic potential. Also, some works have demonstrated the surface of oxide‐derived Cu,^[^ ^10^ ^]^ Zn, and mixed Cu–Zn catalysts^[^ ^11^ ^]^ have been reduced after a few minutes at CO_2_RR‐relevant potentials. For example, Ren et al. employed in situ Raman to probe the surface of the Cu_2_O electrode in real time at −0.99 V, as presented in Figure 2b.^[^ ^10^ ^]^ The vibrational fingerprints of Cu_2_O at 147, 218, 526, and 624 cm^−1^ were detected at the start of the CO_2_ reduction (at 0 s). These peaks weakened after 30 s, demonstrating a rapid reduction of the top layers of the Cu_2_O. Two bands centered at 365 and 502 cm^−1^, attributed to the Cu−O vibrations of intermediately reduced Cu oxides, concurrently appeared. After 200 s, no peaks could be observed in the Raman spectrum. This suggests that the surface of the Cu_2_O has been reduced to metallic Cu during CO_2_ reduction. After the cathodic potential was removed, the surface reoxidized in tens of seconds to Cu_2_O, as shown by the appearance of its Raman bands at 147, 520, and 624 cm^−1^ (the red range). However, Dutta et al. identified SnO_2_, rather than the reduced Sn, as the active surface for CO_2_RR via in situ Raman spectroscopy.^[^ ^12^ ^]^ As shown in Figure 2c, the relative intensities of the A_1g_ peaks in the spectra, attributable to crystalline SnO_2_, are plotted in the right part as a function of the applied electrode potential. Three potential regions labeled from I to III have been distinguished: in region I, the catalyst is present in its native SnO_2_ form; in intermediate region II, the catalyst is partially reduced; in region III, the reduction (to Sn) is already complete. Since the A_1g_ mode of SnO_2_ crystallites exists over 1 h at −1.1 V, the authors assumed SnO_2_, instead of the reduced Sn, is the active species for CO_2_RR. Beyond the studies of catalyst material, works based on carbonate and formate signals have also been published. For example, Schmitt et al. observed the Ag—CO bond on the Ag electrode to become less intense by adding 3,5‐diamino‐1,2,4‐triazole, at the same time the Faradic efficiency (FE) of CO increased.^[^ ^13^ ^]^ Thus, the authors claimed that the addition of 3,5‐diamino‐1,2,4‐triazole could weaken the bond of Ag with CO and enhance the CO FE in electrochemical CO_2_RR.

In summary, in situ Raman spectroscopy studies provide important information regarding the nature of catalytic active sites, which is critical for the identification of the reaction pathway. However, the spatial resolution and lesser sensitivity to the evolution of the catalyst surface restrict it from providing more information. Other techniques are necessary to complement this analytical technique.

### 2.2 X‐Ray Absorption Spectroscopy

Since X‐ray absorption spectroscopy (XAS) can provide information regarding valence electron distribution, X‐ray absorption near‐edge structure (XANES) spectroscopy can reflect the chemical valence states and electronic structure information of the measured elements, while the fitting data from the extended X‐ray absorption fine structure (EXAFS) spectrum reveal the true spatial distribution, bonding conditions, and coordination environment of the atoms.^[^ ^14^ ^]^ Thus, X‐ray based analytical methods are promising for the measurement of structural evolution and real active sites of the electrocatalyst during the CO_2_RR process.

For example, Chen's group monitored the K‐edge of Pd by in situ XANES on a Pd‐loaded carbon catalyst.^[^ ^15^ ^]^ The electrochemical flow cell configuration used for operando X‐ray measurement is shown in Figure 2d. As shown in Figure 2e, both peaks shift to lower energies with increasing overpotentials, which agrees with the reported phenomenon of palladium hydride formation in H_2_ atmosphere. Meanwhile, the Pd–Pd distance gradually increased from 2.732 A (0 V) to 2.842 A (−0.5 V), consistent with bond lengthening due to the formation of PdH. Later, the same group confirmed that a well‐defined PdH phase was not formed until a potential lower than −0.5 V_RHE_ was achieved.^[^ ^16^ ^]^ Based on a previous literature report stating that underpotential deposition of H on Pd could occur at potentials around 0 V_RHE_,^[^ ^17^ ^]^ the authors assumed the presence of surface hydrogen and/or amorphous PdH in the potential range between 0 and −0.5 V_RHE_.

Moreover, in situ EXAFS analysis was performed on Pd‐based alloy (PdAg, PdCu, and PdPt) catalysts.^[^ ^18^ ^]^ It was observed that the presence of Ag or Cu in the Pd lattice did not interfere with the formation of the PdH phase and a longer bond distance between Pd–PdM (M = Ag and Cu) was observed. However, the oxidation state of Pd remained almost unchanged in the applied potential range and the bond lengths of both Pd–Pd and Pd–Pt were the same, confirming that bimetallic PdPt hydride formation was inhibited with the presence of Pt in the PdPt alloy. This may have been because Pt in the PdPt alloy has strong *H binding, causing the formation of the PdPt hydride phase (i.e., H diffusion into lattice) to be unfavorable.

Thus far, nanostructured Cu cathodes have been suggested as the most efficient and selective catalysts for the generation of multicarbon products from the electrochemical CO_2_RR. In 2016, Eilert et al. claimed it was valuable to use oxidized copper precursors for the construction of selective CO_2_RR catalysts and showed that the oxidation state of the precursor did not affect the electrocatalyst selectivity toward ethylene formation.^[^ ^19^ ^]^ However, Jung et al. observed that Cu_2_O nanoparticles reduced to a metallic Cu^0^ state were observed under CO_2_RR, as observed by in situ XANES.^[^ ^20^ ^]^ Moreover, Cu clusters with judiciously controlled surface coordination numbers (CN) from the Hong Kong University of Science and Technology (HKUST‐1) were prepared as a high selectivity, activity, and efficient electrocatalyst for CO_2_RR.^[^ ^21^ ^]^ In situ XAS spectra suggested the formation of Cu clusters with low CN from distorted Cu dimers in HKUST‐1 during CO_2_ electroreduction. Alloy‐based electrocatalysts have been widely investigated because the second metal can affect the interactions of reactants, intermediates, and products at the surface of the catalyst, thereby enhancing or suppressing certain catalytic processes. A bimetallic catalyst of Cu and Co for CO_2_RR was prepared.^[^ ^22^ ^]^ In situ XAS and quasi in situ X‐ray photoelectron spectroscopy (XPS) illustrated that the structure and surface compositions of the bimetallic NPs in as‐prepared states were drastically different from those at working conditions. Specifically, a reduction of both the initially oxidized metallic species and a segregation of the Cu surface was observed under electrochemical conditions.

### 2.3 XPS

XPS can measure the electrons of the materials which ejected by the photoelectrons obtained by the X‐rays, providing data on elemental composition and the chemical and electronic states of the catalyst. However, XPS is typically performed in an ultra‐high vacuum (UHV) environment, creating difficulties for operando electrochemical studies.

Commonly, in situ or quasi in situ XPS is performed by connecting the XPS analysis chamber to an electrochemical cell and allowing sample transfer without exposure to air after the electrochemical reaction has occurred.^[^ ^23^ ^]^ Also, since the measured depths of XPS and XAS are different, these technologies are typically complementary to one another for the study of surface and bulk material properties.

For example, plasma‐oxidized Ag foils show enhanced activity of CO_2_ electroreduction to CO with a 90% FE at −0.6 V_RHE_.^[^ ^24^ ^]^ The operando XAS showed that oxygen species can survive in the bulk of the catalyst during the reaction, but quasi in situ XPS results showed that the catalyst surface is metallic under reaction conditions. Similarly, Cu*_x_*O species were created on dendritic Cu using low‐pressure oxygen plasma.^[^ ^25^ ^]^ The in situ XPS results showed that oxides did not survive during CO_2_RR at an applied potential of −0.9 V_RHE_ (the potential for the maximum production of ethylene), but in situ XAS proved that Cu oxides also exist under these reaction conditions. Even though such oxides might migrate toward the surface during the reaction based on the concentration gradient across the sample, oxidized species went undetected on the surface. Thus, the authors claimed that dendritic morphology plays a superior role in determining their catalytic performance as compared with the initial presence of oxides in the prepared catalysts. It should be noted that the compound catalyst surface was only partially reduced to a metallic moiety during the CO_2_RR, whereas a work based on Zn NPs showed the coexistence of cationic species with a structure resembling that of Zn(OH)_2_ and metallic Zn during CO_2_RR.^[^ ^26^ ^]^

### 2.4 Electron Microscopy

Transmission electron microscopy (TEM) and scanning electron microscopy (SEM) can provide real time data regarding morphological evolution and compositional change of catalyst at the atomic level during the reaction.

Modern in situ liquid phase–TEM (LP‐TEM) holders allow for the electrolyte to flow around the main observation micro‐cell while maintaining compatibility with the UHV chamber. Since it can record atomic and morphological changes almost instantaneously at high resolution, it has been used to study the stability of OER catalysts under electrochemical reaction conditions. For example, an oxygenated species on the surface of Pr_0.64_Ca_0.36_MnO_3_ catalyst was detected and believed to be an OER intermediate.^[^ ^27^ ^]^ In another work, catalyst degradation by dissolution and mechanical dislodgement was observed using in situ LP‐TEM.^[^ ^28^ ^]^ Moreover, this technique can be used to identify catalytically active surfaces. For example, the surface oxidation of Pd nanocrystals was found to first occur at the atomic steps or vertex site, and then proceed to the Pd(111) faces.^[^ ^29^ ^]^ Meanwhile, electrochemical measurements showed that a catalyst rich with these faces also showed significantly higher activity, where Pd(111) was considered as the active face for CO_2_RR. However, it is extremely difficult to perform in situ TEM for CO_2_RR, since the electron beam can induce radiolysis and specimen damage. Moreover, the limited space and positioning of the electrodes inside the TEM holder can cause non‐uniform electric field distribution and create hot spots that artificially enhance electrochemical activity near the electrode tip. As a result, there have been no direct examples of CO_2_RR studies using in situ TEM. It is believed that more effort and research is needed for this endeavor.

Scanning microscopy techniques have been devised to probe surface information of specimens using a fine‐tipped probe able to detect quantum tunneling current. An example is Bao's prepared Au‐loaded CeO_2_ electrocatalyst for CO_2_RR.^[^ ^30^ ^]^ To study the role of the Au−CeO*_x_* interface in CO_2_RR, CeO*_x_* islands were prepared on Au(111) to monitor the interactions between the interface and CO_2_. As shown in Figure 2g, CO_2_ first adsorbs at the interfacial boundaries of Au and CeO*_x_*, and then adsorbs on the surface of CeO*_x_*, but no CO_2_ adsorbs on Au(111). Besides, CO_2_ prefers to bond to previously adsorbed CO_2_ species, forming an adsorbate ring around the edge of the CeO*_x_* island and gradually propagating toward the island center. Also, in situ scanning tunneling microscope (STM) was used to study the origin of the various electrochemical behaviors of metal electrodes. For example, Fu et al. investigated the surface structure evolution of Au single crystals in ionic liquids (ILs).^[^ ^31^ ^]^ Deactivation of Au(111) was observed by electrochemical measurements, while in situ TEM results suggested it to be related to large Au surface restructuring processes taking place in ILs due to the potential‐induced reconstruction and lifting of the surface reconstruction. Moreover, Au(110) was shown to be the most active face, with no regard to the chemical nature of the IL used as the electrolyte, which is consistent with the results of in situ STM showing severe etching on the Au(111) surface at the potentials of CO_2_RR, while the etching process was not observed on Au(110).

In summary, by detecting crystal phase changes, valence state changes, and morphological evolution of catalysts with in situ characterization techniques, the reactive active phase under different conditions can be inferred. Further, combining experiments and theoretical calculations, the mechanism of how catalyst evolution affects the product selectivity and catalytic activity of CO_2_RR could be acquired accurately.

## 3 Metal‐Based Electrocatalysts

Metal electrodes are assumed to be the most popular electrocatalysts for CO_2_RR. Three groups have been roughly classified based on various reaction routes and main products: 1) metals generating HCOOH as the predominant product (Sn and Bi), 2) metals generating CO as the predominant product (Au, Ag, Pd, and Zn), and 3) metals generating hydrocarbons as the predominant product (Cu).^[^ ^32^ ^]^

Sn and Bi metals primarily generate HCOO^−^ in aqueous solution. According to density functional theory (DFT) calculations, the binding energy for *OCHO, which is suggested as the key intermediate for the CO_2_ to HCOO^−^ transformation on Sn and Bi, shows high selectivity.^[^ ^33^ ^]^ Meanwhile, Au, Ag, Pd, and Zn can weakly bond with protons and CO on their surface, which prevents CO from further reduction, hence generating CO as the predominant product. In comparison, Cu is individually divided into a third class, as it favors binding *CO intermediates and converting them into alcohols or other hydrocarbons from *COH or *CHO intermediates through dimerization pathways.^[^ ^34^ ^]^

It is worth noting that other metals like Pt and Ni have lower hydrogen evolution reaction (HER) overpotentials and strong binding capabilities with *CO intermediates. Therefore, HER will be the predominant process in the presence of water.^[^ ^35^ ^]^ However, more reports mention that single‐atom metals, such as Ni and Fe, on carbon present high CO selectivity, suggesting that electrocatalyst design based on classification is not perfect and more definitive correlations should be studied and discovered.

To more accurately predict performance, the concept of electronic structure should be introduced based on the chief principles of catalysis using metallic catalysts. The key factor of the catalytic mechanism is that the interactions between the adsorbate (CO_2_ molecules in this case) and metal surface are largely determined by the d‐band levels of the catalyst itself. Via location adjustment of the d‐band centers, the bonding strengths of adsorbed intermediates (*COOH, *CO, and so on) and Gibbs free energies (Δ*G*) consumed in the rate‐determining steps can be optimized to enhance catalytic performance.^[^ ^8^ ^]^ Hence, achieving good metallic catalyst activities relies on the adjustment of d‐band levels through many approaches, such as particle alloying, heteroatom doping, surface modification, crystal plane/active site exposure (such as terraces, edges, or corners), and so on. Recent and more detailed progress on different classes of metal catalysts for CO_2_ electroreduction is introduced below.

### 3.1 Sn and Bi

**Figure 3:**
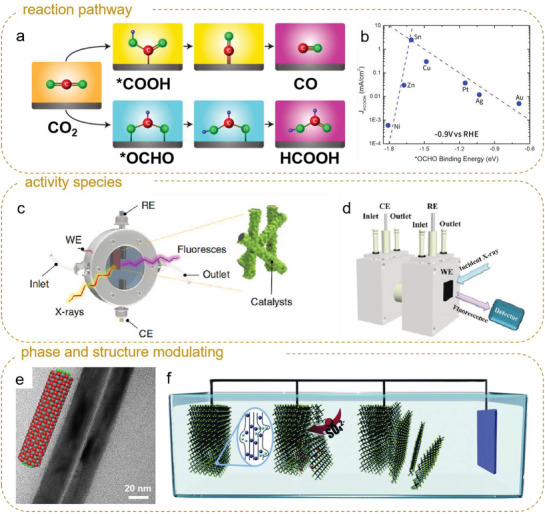
a) Mechanism of CO_2_RR to CO and HCOO^−^. b) Volcano plot using *OCHO binding energy as a descriptor for HCOO^−^ partial current density at −0.9 V_RHE_. Reproduced with permission.^[^ ^33^ ^]^ Copyright 2017, American Chemical Society. c) Schematic illustration of the in situ liquid cell experimental set‐up. Reproduced with permission.^[^ ^37^ ^]^ Copyright 2018, Nature Publishing Group. d) Diagram of the in situ EXAFS liquid cell for CO_2_RR Reproduced with permission.^[^ ^38^ ^]^ Copyright 2019, Nature Publishing Group. e) TEM images and atom model pf CuSn nanowires. Reproduced with permission.^[^ ^40^ ^]^ Copyright 2019, Elsevier. f) Schematic illustration of exfoliated SnS materials dispersed in the solution. Reproduced with permission.^[^ ^42^ ^]^ Copyright 2020, Royal Society Chemistry.

In the 1980s, Hori et al. first reported that several II‐B, III‐A, and IV‐A metals (Pb, Cd, Hg, In, Sn, and Tl) could reduce CO_2_ to formate.^[^ ^32^ ^]^ Unfortunately, many of these heavy metals (Pb, Cd, Hg, and Tl) are highly toxic and environmentally hazardous, which makes them impractical for most applications. Due to the relative abundance of Sn in the Earth's crust and its high selectivity to formate (HCOO^−^) or formic acid (HCOOH), Sn electrodes have attracted considerable attention as candidates for CO_2_RR on a large scale. Jaramilo's group investigated the causes for the high selectivity of formic acid by polycrystalline Sn surfaces and performed theoretical calculations.^[^ ^33^ ^]^ The authors proposed two pathways of production for CO and HCOOH in **Figure** **3**a. The DFT results show the binding energy for *OCHO on Sn, which is suggested to be the key intermediate for the CO_2_ to HCOO^−^ transformation, is on the top of the volcano plot (Figure 3b). Thus, it has a high selectivity for HCOO^−^. Based on these calculations, the scientists sought to develop more highly efficient electrocatalysts.

It is well known that nanostructured catalysts typically exhibit superior electrochemical activities due to more active sites compared with bulk catalysts. However, the smaller the nanomaterial, the easier it is to be oxidized in air. To solve this issue, Xie's group reported that graphene‐confined Sn quantum sheets (Sn@G) showed a FE value of 89% at a potential of −1.0 V_RHE_. Electrochemical measurements revealed that the Sn@G electrode provided nine times higher CO_2_ adsorption capacity, and the OH^−^ adsorption potential of Sn@G was 0.13 V lower than that of bulk Sn electrode.^[^ ^36^ ^]^

Even though Sn electrodes show high selectivity in formate, it has only been achieved at highly cathodic potentials. SnCu alloys are typically prepared because Cu is an inert electrocatalyst for HER and induces strong binding of *CO intermediates, which can suppress the HER and production of CO. Work from Cui's group demonstrated that Sn in a CuSn_3_ alloy has an oxidation state of Sn*^*δ*^* ^+^ at −0.5 V_RHE_.^[^ ^37^ ^]^ However, pure Sn is a zero‐valence material with no changes in oxidation state at the same potential, as proven by in situ XAS (Figure 3c). It agrees with the theoretical results that Sn donates 0.1 electrons to Cu during the CO_2_RR process. Later, another work from Wang's group supported this same conclusion. As shown in the Sn K‐edge EXAFS spectra, the peak that indexes to Sn—O bonding is observed in both Sn and SnCu at open circuit potentials (Figure 3d).^[^ ^38^ ^]^ However, the Sn—O bonding in Sn will disappear, while the bond in the SuCu alloy is still present, indicating that the real active species of Sn is the oxidized Sn.

The geometric structure of the alloy will also affect electrocatalytic performance. For example, Hou et al. prepared a 3D core–shell porous Cu@Sn electrocatalyst. The optimized presence of granularly structured Sn on the Cu surface was found to be a key factor for enhancing HCOOH selectivity and mass activity.^[^ ^39^ ^]^ Huang's group prepared a SnCu nanowire (NW) and modulated the electronic interactions between the Cu and Sn species by controlling the thermal annealing in different atmospheres, such as O_2_, N_2_, and H_2_ (Figure 3e).^[^ ^40^ ^]^ The surface valence band photoemission spectra showed the d‐band center of different CuSn NW/C shifting toward Fermi levels after the introduction of the Sn species compared with Cu NWs/C, indicating stronger binding strengths to the HCOO* intermediate. The authors also applied first‐principle calculations to reveal the origin of the enhanced electrocatalytic performance. It was claimed that Sn atoms doped into the CuO(111) surface enhanced the adsorption of the CO_2_ intermediate *OCHO and suppressed H_2_ production, thus realizing high selectivity on CuSn NWs/C‐Air.

Metal electrode preparation from oxides is the most commonly used strategy because of the convenience and ability to control morphology. Moreover, it is claimed that the moderate content of heteroatoms, such as O and S, can change the electronic structure of the catalyst and create sites with higher CO binding energy, thus enhancing CO_2_RR performance. For instance, Lou et al. studied the correlation between grain boundaries and the enhanced reactivity in catalytic activity with sub‐2 nm SnO_2_ quantum wires (QWs) as a model electrocatalyst.^[^ ^41^ ^]^ With more exposed grain boundaries, the ultrathin SnO_2_ QWs showed significantly higher current density and improved FE (over 80% for HCOOH and ≈90% for C_1_ products) and energy efficiency (over 50%) as compared with SnO_2_ nanoparticles. Recently, ultrathin SnS nanosheets with a thickness of 5 nm and lateral size of 1 µm were prepared using an electrochemical exfoliation strategy (Figure 3f).^[^ ^42^ ^]^ The electrocatalyst exhibited an FE of 82.1% to formate and practical current density of 18.9 mA cm^−2^ at −1.1 V. The efficient catalytic activity was ascribed to the synergistic effects between the ultrathin layer‐induced defects and exposed (001) plane of SnS NSs. This research on Sn electrodes may provide a feasible pathway for the enhanced yield of HCOOH through the optimization of electrocatalysts. However, the overpotential is still quite high, typically higher than −0.8 V_RHE_, and the current density is still lower than that required by industry.

The possible intermediates in the CO_2_RR process are *H, *COOH, and *OCHO. According to DFT calculations, the binding strengths of these three intermediates on Sn are reported to be relatively comparable, which causes inferior formate selectivity.^[^ ^43^ ^]^ On the contrary, CO_2_RR to formate on bismuth (Bi) is the most energetically favorable among the three competing cathodic processes. Thus, Bi electrocatalysts mainly produce formate while inhibiting other processes. As listed in **Table** **2**, all Bi nanosheet electrocatalysts show high selectivity for formate. Four types of active sites for CO_2_RR on Bi catalysts have been reported: the edge and corner, lattice planes, defects, and interfaces.

**Table 2:** The recently reported metal electrocatalysts produce HCOOH and HCOO^‐^ as the main product for electrochemical CO_2_RR

Electrocatalyst	Faradaic efficiency [%]	Potential [V_RHE_]	*j*_product_ [mA cm^−2^]	Electrolyte	Ref.
polycrystalline Sn	70	−0.9	10	0.1 m KHCO_3_	^[^ ^32^ ^]^
Graphene confined Sn sheets	89	−0.8	21.2	0.1 m NaHCO_3_	^[^ ^36^ ^]^
CuSn_3_	95	−0.5	33	0.1 m KHCO_3_	^[^ ^37^ ^]^
Sn‐Cu/SnO*_x_* core/shell	83	−0.93	406.7	0.5 m KHCO_3_	^[^ ^38^ ^]^
Cu@Sn core shell	100	−0.93	16.52	0.5 m KHCO_3_	^[^ ^39^ ^]^
CuSn nanowires	90.2	−1.0	17.33	0.5 m KHCO_3_	^[^ ^40^ ^]^
SnO_2_ quantum wires	80	−1.2	18	0.1 m KHCO_3_	^[^ ^41^ ^]^
SnS nanosheet	82.1	−1.1	18.9	0.5 m KHCO_3_	^[^ ^42^ ^]^
Bi nanosheet	95	−1.5	11	0.5 m NaHCO_3_	^[^ ^43^ ^]^
Bi nanoflake	79.5	−0.4	‐	0.1 m KHCO_3_	^[^ ^44^ ^]^
Bi nanosheet	86	−1.1	16.5	0.1 m KHCO_3_	^[^ ^45^ ^]^
Bi nanotube	97	−1.1	39.4	0.5 m KHCO_3_	^[^ ^46^ ^]^
Bi nanosheet	100	−0.9	7	0.5 m NaHCO_3_	^[^ ^47^ ^]^
Bi nanosheet	95	−1.16	57	0.5 m KHCO_3_	^[^ ^48^ ^]^
Sulphide derived Bi	84	−0.75	5	0.5 m NaHCO_3_	^[^ ^49^ ^]^
Defective Bi nanotube	93	−1.0	60	0.5 m KHCO_3_	^[^ ^50^ ^]^
BiSn	96	−1.1	50	0.5 m KHCO_3_	^[^ ^51^ ^]^
Bi‐ene	98.6	−0.9	30	0.5 m KHCO_3_	^[^ ^52^ ^]^

**Table 3:** The recently reported metal electrocatalysts produce CO as the main product for electrochemical CO_2_RR

Electrocatalyst	Faradaic efficiency [%]	Potential [V_RHE_]	*j*_product_ [mA cm^−2^]	Electrolyte	Ref.
Ag nanocubes with length of 25 nm	99	−0.856	3	0.1 m KHCO_3_	^[^ ^57^ ^]^
Pd octahedra particles	85	−0.9	41	0.5 m NaHCO_3_	^[^ ^56^ ^]^
Porous Zn	95	−0.95	270	0.1 m KHCO_3_	^[^ ^58^ ^]^
Zn NPs	84.4	−0.7	4	0.5 m KHCO_3_	^[^ ^59^ ^]^
Ligand modified Ag NPs	93	−0.6	1.9	0.1 m NaHCO_3_	^[^ ^60^ ^]^

**Figure 4:**
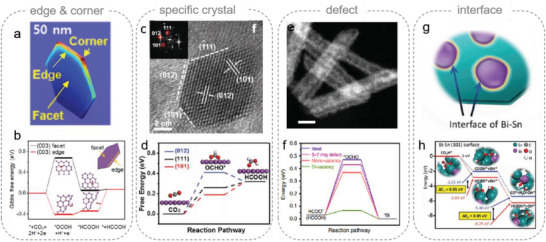
a) Simulated electric field distribution in 3D Bi nanostructures; Reproduced with permission.^[^ ^44^ ^]^ Copyright 2017, Elsevier. b) DFT‐calculated Δ*G* in the reaction pathways of CO_2_ conversion into formate from the facet sites and edge sites of (003) plane on Bi. Reproduced with permission.^[^ ^45^ ^]^ Copyright 2017, Elsevier. c) TEM images at different magnifications, and corresponding SAED pattern of reduced Bi nanosheets; d) Free‐energy diagram for HCOOH on Bi (101), (111), and (012) planes. Reproduced with permission.^[^ ^47^ ^]^ Copyright 2018, Wiley‐VCH. e) STEM‐HAADF images of Bi_2_O_3_ NTs. f) Free‐energy profiles for formate production on ideal and defective Bi surfaces. Reproduced with permission.^[^ ^50^ ^]^ Copyright 2019, Nature Publishing Group. g) Schematic illustration of the interface of Bi‐Sn; h) Calculated reaction energy profiles for CO_2_RR to form CO (top) and HCOOH (bottom) on the Bi‐Sn (101) surface. Reproduced with permission.^[^ ^51^ ^]^ Copyright 2018, Wiley‐VCH.

The edge and corner sites are commonly considered as active sites in CO_2_RR. For example, Kim et al. designed Bi nanoflakes with several edges and corners on the Cu substrate using a novel pulse electrodeposition method.^[^ ^44^ ^]^ They used COMSOL multiphysics simulation to explore the prospects of edge‐ or corner‐enhanced nanometer‐scale field intensification, which could enhance the electrocatalytic performance of Bi nanoflakes with abundant edges and corners (**Figure** **4**a).^[^ ^44^ ^]^ Later, Jin's group proposed a sonication–exfoliation strategy to prepare Bi nanosheets with a thickness of 1–2 nm.^[^ ^45^ ^]^ DFT calculations revealed that the *OCHO formation step tended to occur on edge sites rather than on facet sites via DFT calculations (Figure 4b). Recently, Fan et al. claimed the limiting potential for CO_2_ reduction to HCOOH decreased with increasing curvature.^[^ ^46^ ^]^ They suggested a broad potential window for formate formation on Bi could be achieved using nanotube structure design. Utilizing this discovery, Bi nanotubes with highly curved surfaces and rich edges were prepared, showing selectivities >80% in a significantly larger potential window of about 600 mV.

Moreover, some specific lattice planes were found to achieve higher activity than other planes. A DFT calculation from Lu's group suggested that the (101) and (111) planes of Bi could significantly stabilize *OCHO and, therefore, were the most active sites for formate production (Figure 4c,d).^[^ ^47^ ^]^ Besides theoretical calculations, in situ XAFS was applied to investigate the origin of improved catalytic activity.^[^ ^48^ ^]^ A shortened inter‐layer Bi—Bi bond length, which indicates p‐orbital delocalization, was observed in the reduced Bi nanosheet. The authors assumed that modulating the electron density of localized p‐orbitals in Bi into delocalized states could provide versatile electronic structures and enhance the reduction activity to produce formate.

Defects on the surfaces of Bi‐based catalysts have been considered as active sites. For example, Zhang et al. reported that defect‐rich sulfide‐derived Bi was an electrocatalyst.^[^ ^49^ ^]^ By comparing the electrocatalytic performances with/without lattice disorder, the authors claimed that the lattice defects, rather than the residual sulfur, were likely to stimulate a positive effect on the catalytic reduction of CO_2_. This study agrees with other work from Li's group. A structurally defective Bi nanotube was prepared by electrochemical reduction, and DFT calculations reveal the ΔG for formation of *OCHO was reduced from +0.47 eV on ideal Bi (001) surfaces to +0.07 eV on Bi surfaces with vacancies, indicating that *OCHO intermediates could be stabilized by abundant defective sites on the obtained electrocatalyst (Figure 4e,f).^[^ ^50^ ^]^

Also, the interface between two crystals has been claimed to be an active site. For example, Wen et al. reported a Bi–Sn alloy electrocatalyst with a formate FE of 96% at −1.14 V_RHE_.^[^ ^51^ ^]^ As shown in Figure 4g, the Sn–Bi interface induced active sites via favorable orbital interactions, where the Bi–Sn bimetallic catalyst converted CO_2_ to formate with remarkably high FE (96%) and production rate (0.74 mmol h^−1^ cm^−2^) at −1.1 V_RHE_. DFT calculations showed shifted peaks of both Bi and Sn, indicating electron transfer from Sn to Bi atoms. The projected density of states of the surface Sn atoms on the Sn (101) and Bi–Sn (101) surfaces were analyzed by deconvoluting the electron density and wave function into atomic orbital contributions. Both the p and d orbitals of Sn electron states upshifted away from the Fermi energy level after interactions with Bi. Thus, electrons from more electronegative O atoms were readily transferred to the p and d orbitals of Sn atoms, enhancing the adsorption energy of the *OCHO intermediate to the Bi–Sn (101) surface, which led to improved selectivities of CO_2_RR toward formate production (Figure 4h).

There have been active studies attempting to increase active sites by increasing surface area. Recently, an atomically thin bismuthene (Bi‐ene) was pioneered via an in situ electrochemical transformation process from ultrathin bismuth‐based metal‐organic layers.^[^ ^52^ ^]^ The few‐layer Bi‐ene exhibited high selectivity of formate (≈100%), large partial current density (72 mA cm^−2^), and good stability (FE = 98.6% after 12 h) over a wide potential window exceeding 0.35 V. Interestingly, the in situ attenuated total reflection infrared (ATR‐IR) and DFT results confirmed that the adsorbed HCO_3_^−^ groups from the electrolyte played important roles during CO_2_RR. Some of the easily absorbed HCO_3_^−^ groups could directly participate in formate generation when the applied overpotential was low, whereas formate was primarily generated by the reduction of the CO_2_ molecules from the feed gas, as well as the dissociation of the adsorbed HCO_3_^−^ groups at higher potential. It is worth noting that product selectivity is a complicated process; thus, the reduced products of CO_2_ on Bi‐based electrocatalysts could be CO,^[^ ^53^ ^]^ methanol,^[^ ^54^ ^]^ or other chemicals, depending on the surface conditions and electrode composition.

In summary, several investigations of active sites have been carried out. Meanwhile, Sn‐ and Bi‐based electrocatalysts with high selectivity have been reported by rational design, such as the introduction of a second metal, defect creation, and specifically‐designed morphology preparation. However, the potential of the reduction reaction is still much higher than the theoretical value, and most investigations regarding the adsorption sites of *OHCO intermediates are based on theoretical calculations, which usually differ from the actual reaction process. It is pivotal to monitor the electrocatalyst to determine the real reactive sites and rationally design an electrocatalyst with high FE over a full potential window.

### 3.2 Pd, Zn, and Ag

In 1994, Hori et al. demonstrated that second group metals, Au, Ag, Pd, and Zn, used as catalysts for CO_2_RR predominantly yielded CO.^[^ ^32^ ^]^ These metals can weakly surface‐bond with protons and CO and prevent CO from further reduction, while generating CO as the predominant product (**Table** **3**).^[^ ^55^ ^]^

**Figure 5:**
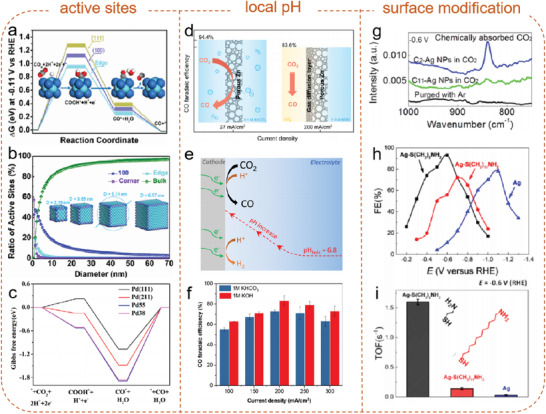
a) Free energy diagrams for CO_2_RR to CO on edge, Ag(111), and Ag(100) at −0.11 V; b) percentages of different active sites on Ag NCs as a function of diameter. Reproduced with permission.^[^ ^57^ ^]^ Copyright 2016, Wiley‐VCH. c) Free energy diagrams for CO_2_ reduction to CO on Pd (111), Pd (211), Pd55, and Pd38. Reproduced with permission.^[^ ^56^ ^]^ Copyright 2013, American Chemical Society. d) The comparison of current density and CO FE between porous Zn and gas diffusion layer supported porous Zn; e) Schematic illustration of local pH effect in CO2RR; f) CO FE as a function of geometric current density. Reproduced with permission.^[^ ^58^ ^]^ Copyright 2019, American Chemical Society. g) In situ ATR‐IR spectra, h) CO FE, and i) TOF for CO at −0.6 V of Ag, C_2_−Ag, and C_11_−Ag NPs. Reproduced with permission.^[^ ^60^ ^]^ Copyright 2016, American Chemical Society.

In 2015, Wang's group discovered that corner and edge sites on Pd nanoparticles were more active than terrace sites toward CO_2_RR, whereas the formation of H* for HER was similar on all three sites.^[^ ^56^ ^]^ A volcano‐like curve of the turnover frequency for CO formation implied that the formation of HCOO* and CO* began to dominate the reaction rate. Furthermore, *CO removal during the CO_2_RR process can be tailored by controlling the size of Pd NPs due to the changing ratios of corners, edges, and terrace sites. Similarly, Luo's group claimed the catalytic activity of CO formation on Ag nanocubes could be influenced by the density of catalytically active edges and Ag (100), which was controllable by the particle size and surface structure.^[^ ^57^ ^]^ DFT calculations were performed to calculate a free energy diagram based on the computational hydrogen electrode model. Compared with Ag (111), the required Δ*G*s to generate COOH* and CO* on Ag (100) and edge sites were remarkably lower (**Figure** **5**a,b). Besides, Ag (100) and the edge showed comparably lower work functions and smaller d‐band centers, which is conducive to faster electron transfer and stronger binding abilities to intermediates. Recently, Zhu et al. synthesized nanosized Pd octahedral particles dominated by Pd (100) and Pd (111) facets.^[^ ^16^ ^]^ DFT calculations showed that the reduced binding energies of CO and HOCO intermediates on PdH (111) were critical parameters to the high current density and FE for CO_2_ to CO conversion (Figure 5c). Also, in situ XAS studies showed that Pd was transformed into Pd hydride (PdH) when the potential was lower than −0.5 V_RHE_. Thus, the Pd particles showed a high CO FE of 95% at a potential of −0.5 V_RHE_.

Morphological engineering is an efficient way to create edges, corner sites, or specific facets as active sites to enhance electrocatalytic activity. Also, it can influence the valence states of metal atoms and local pH. In 2018, work from Cuenya's group confirmed that the oxidation state of Zn during CO_2_RR was dependent on particle size by in situ XAFS. It indicated that the high selectivity of Zn NPs was derived from the structure or morphology of the Zn NPs and the stabilization of cationic Zn species under reaction conditions. In another work, a porous Zn electrocatalyst was prepared by electrodeposition.^[^ ^58^ ^]^ By comparing the electrocatalytic activities of porous Zn electrodes with different Zn loadings and surface areas, the author claimed that the porous structure dramatically increased the active site number and induced high local pH (Figure 5d–f), thus boosting the performance of CO_2_RR.

Also, surface modifications of metal electrodes with molecular ligands and surfactants have been studied. For example, an *N*‐heterocyclic carbene‐functionalized approach was reported to tune Au NPs electrocatalysts for CO_2_RR.^[^ ^59^ ^]^ The strong *σ*‐donation from the carbenes enabled the Au NPs surface to be highly electron‐rich, so a fast electron transfer to CO_2_ occurred before the rate‐determining step, which was evidenced by the Tafel slope decreasing from 138 to 72 mV dec^−1^. Besides electron transfer, other groups found the surface ligand (—S—CH_2_—CH_2_—NH_2_) could bond with molecular CO_2_ by in situ ATR‐IR spectroscopy.^[^ ^60^ ^]^ The chemical interactions between NH_2_ and CO_2_ facilitated CO_2_ activation and led to a 0.3 V decrease in overpotential for CO formation (Figure 5g–i). However, the ligand‐assisted chemisorption of CO_2_ on Ag catalysts did not occur when the number of carbons was 11 in the ligands (C_11_‐Ag NPs).

It is worth noting that the CO_2_RR process is complicated, and the main product can be affected by many factors; thus, the obtained product is adjustable by the electronic structure and surface microenvironment. For instance, Dutta et al. prepared a Ag foam catalyst, which yielded CH_4_ as the reaction product at potentials more negative than −1.1 V_RHE_ and reached a maximum FE of 51% at −1.5 V_RHE_ before it decreased again.^[^ ^61^ ^]^ It is the first reported CO_2_RR catalyst, other than Cu, that has demonstrated a remarkably high selectivity toward hydrocarbon formation.

### 3.3 Cu

Cu is a promising electrocatalyst for CO_2_ conversion since it is the only metal known to catalyze CO_2_ to hydrocarbons with significant yields.^[^ ^62^ ^]^ Due to its unique properties, many reviews based on Cu have been published. For example, Sun's group summarized the morphology, size, alloy, and support effects with respect to electrocatalytic performance.^[^ ^63^ ^]^ Lee et al. also summarized a review on a Cu‐based alloy.^[^ ^64^ ^]^ They focused on possible phase segregation with concurrent compositional changes and easily oxidized surface‐exposed metals. Qiao's group categorized the secondary metal in Cu‐based alloys by O and H affinities. It linked the effects of extrinsic chemical composition and physical structure to intrinsic intermediate adsorption with reaction pathway selection.^[^ ^65^ ^]^ Besides the electrocatalytic performance of Cu as an electrocatalyst, the effects of Cu on electrolytes have also been discussed.^[^ ^66^ ^]^ Considering that there are already many reviews in this field, we will only briefly introduce Cu‐based catalysts in this review. The main products of recently reported Cu‐based catalysts are listed in **Table** **4**. For each type of catalysts, scientists have made significant efforts to determine the real catalytic sites during the CO_2_RR process.

**Table 4:** Comparison of CO_2_RR Products from various recent reported Cu‐based catalysts

Sample	E [V_RHE_]	Main product	FE [%]	*j*_product_ [mA cm^−2^]	Ref.
Electroredeposited Cu	−1.2	C_2_H_4_	40	22	^[^ ^67^ ^]^
CuB NPs	−1.1	C_2_H_4_	52	70	^[^ ^68^ ^]^
Iodine‐modified Cu	−0.9	C_2+_	31.2	80	^[^ ^69^ ^]^
Cu nanocube on Cu foil	−1.05	C_2_H_4_	25	‐	^[^ ^70^ ^]^
Cu@CeO_2_	−1.2	CH_4_	54	0.5	^[^ ^71^ ^]^
Oxygen plasma‐modified dendrites Cu	−1.2	C_2_H_4_	54	31	^[^ ^25^ ^]^
Cu_2_O NP/C	−1.1	C_2_H_4_	57.3	21	^[^ ^20^ ^]^
HQ‐Cu	−1.05	C_2+_	68.2	45	^[^ ^72^ ^]^
Cu nanocube	−1.012	C_2_H_4_	60	40	^[^ ^73^ ^]^
Cu particles with atomic scale spacings	−0.9	C_2+_	75	8	^[^ ^74^ ^]^
Metal−Organic Frameworks Mediate Cu Coordination	−1.07	C_2_H_4_	45	262	^[^ ^21^ ^]^
Star decahedron Cu NPs	−1.0	C_2_H_4_	52.43	2	^[^ ^75^ ^]^
Cu loaded on PTFE (75% CO_2_)	‐	CH_4_	48	108	^[^ ^78^ ^]^

**Figure 6:**
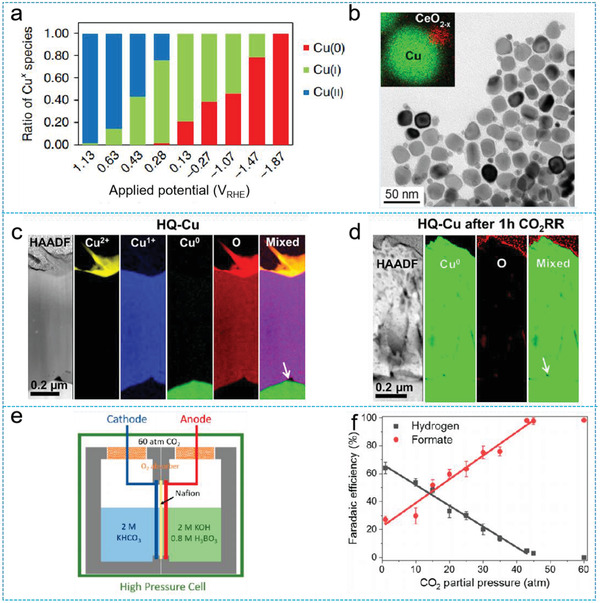
a) Calculated ratio of Cu oxidation states from linear combination fitting vs. applied potential; Reproduced with permission.^[^ ^67^ ^]^ Copyright 2018, Nature Publishing Group. b) TEM image of Cu/CeO_2−_ *_x_* and EDX elemental maps. Reproduced with permission.^[^ ^71^ ^]^ Copyright 2019, American Chemical Society. c,d) High‐angle annular dark‐field STEM image and corresponding EELS mapping on different Cu oxidation states and oxygen of the focused ion beam‐fabricated specimen that corresponds to the cross section of HQ‐Cu and HQ‐Cu after 1 h of the CO_2_RR at −1.05 V_RHE_. Reproduced with permission.^[^ ^72^ ^]^ Copyright 2020, American Chemical Society. e) Schematic of the two‐electrode high‐pressure full cell. f) Dependence of the formate and H_2_ formation FEs on the CO_2_ partial pressure at −0.64 V_RHE_, showing the formate increase and HER decrease with increasing pressure to 45 atm. Reproduced with permission.^[^ ^79^ ^]^ Copyright 2020, American Chemical Society.

It is widely accepted that Cu^+^ species play a critical role in the selectivity toward C_2+_ products. Utilizing in situ XAS, Cu^+^ species were found to exist at a negative potential of −1.47 V_RHE_ under CO_2_RR conditions, which is the primary potential window of C_2+_ products (**Figure** **6**a).^[^ ^67^ ^]^ Thus, it is believed that the existence and stabilization of Cu^+^ at negative potentials usually lead to high selectivities toward C_2+_ products. To tailor the oxidation state of Cu species at the working potential and improve the stability of oxidized Cu, researchers have developed many novel strategies. For instance, Sargent's group modified the local electronic structure of Cu with positive valence sites (boron doping) to boost the conversion of CO_2_ to C_2_ products.^[^ ^68^ ^]^ Experimental results showed that doped boron can increase the percentage of Cu*^*δ*^* ^+^ and improve the stability and C_2_‐product generation. Simulations showed that controlling the average oxidation state of Cu can tune CO adsorption/dimerization, resulting in a preference for C_2_ products. Similarly, halides from electrolytes can modify the surface of Cu electrocatalysts. Cuenya's group reported that an iodine‐modified catalyst displayed a high FE of 80% and a partial current density of 31.2 mA cm^−2^ for C_2+_ products at −0.9 V_RHE_.^[^ ^69^ ^]^ In situ XAS and XPS measurements revealed that the high C_2+_ selectivity of these nanostructured Cu catalysts could be attributed to the highly roughened surface morphology induced by the synthesis and presence of subsurface oxygen and Cu^+^ species and the adsorbed halides from the electrolyte.

Supports can also affect the chemical state of Cu electrocatalysts during CO_2_RR. To study the effects of supports, Grosse et al. prepared two Cu nanocubes loaded on carbon and Cu foil, respectively.^[^ ^70^ ^]^ In situ XPS revealed that interactions between Cu cube/Cu foil stabilized the Cu^+^ species during CO_2_RR, leading to enhanced CO_2_RR electrocatalytic activity. In another study by Varandili et al., CeO_2_ was used as a support for CO_2_RR (Figure 6b).^[^ ^71^ ^]^ In situ XAS confirmed the existence of the partial reduction of Ce^4+^ to Ce^3+^ under standard working conditions. DFT calculations proposed unique active sites formed at the interface could stabilize CO_2_RR intermediates by bidentate adsorption at both Cu and O‐vacancy sites of CeO_2−_ *_x_*, a configuration that allows one to break the CHO*/CO* scaling relationship limitations.

Interestingly, even though many works have claimed that the oxidation state of Cu species is vital for good CO_2_ selectivity, other researchers have found that the morphology of the electrocatalysts, rather than the Cu_x_O species, determines the level of CO_2_ conversion. For example, Scholten et al. prepared a dendritic Cu catalyst by oxygen plasma treatment.^[^ ^25^ ^]^ It has been shown that copper oxides were reduced to metallic Cu at a potential of −0.9 V_RHE_, which is the peak performance potential for ethylene production, indicating that overall catalyst morphology plays a more important role than the initial presence of oxides. Later, Jung et al. prepared Cu_2_O nanoparticles by electrochemical fragmentation.^[^ ^20^ ^]^ They found these nanoparticles (NPs) were transformed into small, fragmented NPs under the proposed reaction conditions. The smaller NPs were reduced to metallic Cu during the CO_2_RR process and prone to reoxidation at open circuit potential inside the electrolyte, yielding labile Cu states. Hence, the authors attributed the improved electrocatalytic performance to the unique morphology, small size, and compact arrangement. Coincidentally, Lei et al. prepared two Cu‐based electrodes with mixed oxidation states, namely, HQ‐Cu (containing Cu, Cu_2_O, CuO) and AN‐Cu (containing Cu, Cu(OH)_2_).^[^ ^72^ ^]^ They found that both electrodes were reduced to Cu^0^, indicating that high C_2+_ selectivities were not associated with the oxidation states of Cu (Figure 6c,d). Meanwhile, the oxide/hydroxide crystals in HQ‐Cu and AN‐Cu were fragmented into nanosized irregular Cu grains under the applied negative potentials. This fragmentation process not only built an intricate network of grain boundaries but also exposed a variety of high‐index facets. These two features greatly facilitated C—C coupling and accounted for enhanced C_2+_ selectivity.

Besides the overall morphology, some particular facets were proven to favor C_2+_ product formation over others. For example, Jiang et al. predicted the initial C‐C coupling steps on Cu(100) and stepped (211) facets preferred C_2+_ product formation to Cu(111).^[^ ^73^ ^]^ Experimentally, they tuned the facet exposure on Cu foil through a metal ion battery cycling method. Compared with polished Cu foil, the cycled Cu nanocube catalyst with exposed (100) facets presented a sixfold improvement in C_2+_ to C_1_ product ratio. It exhibited a high C_2+_ FE over 60% and H_2_ below 20%, and a corresponding C_2+_ current of more than 40 mA cm^−2^. Similarly, Cu particles with various atomically‐scaled spacings between two facets were created by lithiation.^[^ ^74^ ^]^ In situ XAS showed that oxidized Cu was reduced to a metallic state during the CO_2_RR. Meanwhile, facet spacing maximized the binding energies of CO_2_RR intermediates and boosted C—C coupling reactions, leading to high activity and C_2+_ selectivity. Finally, the defects on the Cu surface could be tuned and affect the electronic structure of Cu surface and adsorption of intermediates. It was confirmed that under‐coordinated sites^[^ ^21^ ^]^ and boundaries^[^ ^75^ ^]^ have a positive role in enhancing electrocatalytic activity.

Molecular modification is an effective way to improve catalytic activity. Organic molecules neighboring heterogeneous active sites provide additional binding interactions that can control intermediate stability by improving catalytic performance through increased FE (product selectivity), as well as decreasing overpotential.^[^ ^76^ ^]^ Recently, it was reported that the FE of C_2+_ hydrocarbons on a Cu surface can increase from ≈15% to 60% at −1.1 V_RHE_ by coating a 50 nm film of polyaniline.^[^ ^77^ ^]^ The in situ infrared spectroscopy results showed the improved properties were due to the improved coverage and interactions of the CO intermediate, which facilitated CO−CO coupling.

Interestingly, researchers found that electrocatalytic performance can be enhanced by increasing the concentration of CO_2_ in the electrolyte. For example, the selectivity of methane production can be increased by tuning the concentration of CO_2_ in the gas stream, which can control the local CO_2_ availability on Cu catalysts.^[^ ^78^ ^]^ DFT calculations reveal that lowering *CO_2_ coverage on the Cu surface decreases the coverage of the *CO intermediate and favors the protonation of *CO to *CHO, a key intermediate for methane generation, compared with the competing step, C−C coupling. As a result, (48 ± 2)% methane FE and a methane cathodic energy efficiency of 20% were achieved with a partial current density of (108 ± 5) mA cm^−2^ using a dilute CO_2_ gas stream. Similarly, high selectivity of CO_2_ reduction to formate was achieved using a high‐pressure electrolysis cell (Figure 6e,f).^[^ ^79^ ^]^ The authors claimed that the high‐pressure conditions significantly increased the solubility of CO_2_ and suppressed HER.

Recently, a branched CuO was prepared by controlled oxidation with aqueous NH_3_ as a highly selective electrocatalyst for ethylene production (over 70%).^[^ ^80^ ^]^ Compared with cubic morphology, the initial branched structure formed highly active domains with interfaces and junctions in‐between during activation, which led to large surface areas with high local pH, resulting in high selectivity and activity for ethylene production. Separate from morphology control, metal compounds have been applied to regulate the valence states of metal. Sargent's group prepared a copper on Cu^+^ composite (Cu@Cu_3_N), which stabilized the Cu^+^ during reduction through the use of Cu_3_N as an underlying Cu^+^ species.^[^ ^81^ ^]^ Compared with a pure Cu sample, the Cu@Cu_3_N electrode exhibited a 40‐fold enhancement of selectivity of C_2+_ to the competing CH_4_. Recently, Hod's group prepared a Cu_2_O@CoS_x_ electrocatalyst using an electrochemically driven cation exchange method.^[^ ^82^ ^]^ The rich grain boundaries and under‐coordinated sites acted as active species during CO_2_RR, exhibiting a CO_2_‐to‐formate FE greater than 87%.

Although many efforts have been made, a decisive and precise factor related to the selectivity of a particular product is still being debated. However, it is known that Cu‐based catalysts suffer from high overpotential, low selectivity, and competitive HER reactions. The origin of the poor selectivity is the moderate binding energy of Cu on most reaction intermediates and the adsorption energies of different intermediates, because they relate to one another; thus, it is difficult to tailor the binding of one specific intermediate without impacting another.

## 4 Carbon‐based Electrocatalysts

### 4.1 Metal‐Free Carbon Electrocatalysts

Although an increasing number of metal heterocatalysts have been studied for CO_2_RR applications, most of them still suffer from apparent drawbacks, such as high cost and undesirable and irreversible HERs. Since 2017, many literature articles have demonstrated that metal‐free carbon electrocatalysts have the ability to reduce CO_2_ to CO (**Table** **5**).^[^ ^83^ ^]^ However, pristine carbon is often electrochemically inert toward the CO_2_RR because neutral carbon atoms have negligible ability to activate the CO_2_ molecule. Thus, heteroatom (e.g., F, N, or S) incorporation and functional group modification have often been used to enhance the electrochemical activity of pristine carbon.^[^ ^84^ ^]^ Besides, nanostructured carbon materials like porous carbon, carbon fibers, carbon nanotubes, and graphene have been considered as potential alternatives due to decent catalytic activity, long durability, and high selectivity.

**Table 5:** The recently reported metal‐free carbon electrocatalysts for electrochemical CO_2_RR

Electrocatalyst	Faradaic efficiency [%]	Potential [V_RHE_]	*j*_product_ [mA cm^−2^]	Electrolyte	Ref.
F‐doped carbon	89.6	−0.6	0.24	0.1 m NaClO_4_	^[^ ^85^ ^]^
F‐doped carbon cage	88.3	−1.0	37.5	0.5 m KHCO_3_	^[^ ^86^ ^]^
N‐doped porous carbon	98.4	−0.55	3.2	0.5 m KHCO_3_	^[^ ^87^ ^]^
Se‐doped porous carbon	90	−0.6	9	0.1 m KHCO_3_	^[^ ^88^ ^]^
N, S co‐doped porous carbon membranes	94	−0.7	103	0.1 m KHCO_3_	^[^ ^89^ ^]^
Aziridine‐functionalized CNTs	88	−1.2	0.27	0.1 m KHCO_3_	^[^ ^90^ ^]^
Carboxyl group functionalized graphene	86	−0.68	3	0.5 m KHCO_3_	^[^ ^91^ ^]^

**Figure 7:**
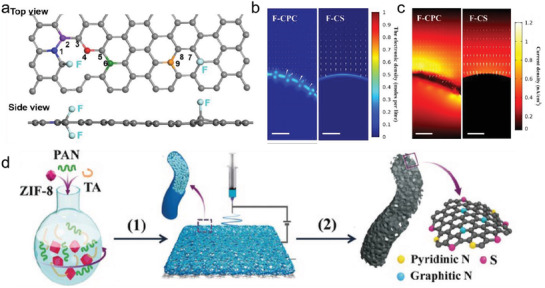
a) Top view and side view of DFT model for F‐doped carbon. Gray atom: carbon, light blue atom: fluorine, other colorful atoms: carbon that calculated as active sites. Reproduced with permission.^[^ ^85^ ^]^ Copyright 2018, Wiley‐VCH. b) Computed electric field and free electron density distributions and c) Current density distributions during CO_2_RR on the surface of F‐doped cagelike porous carbon (F‐CPC) and F‐doped carbon sheet (F‐CS). Scale bars represent 20 nm. Reproduced with permission.^[^ ^86^ ^]^ Copyright 2018, American Chemical Society. d) Synthesis of NSHCF: (1) electrospinning of polymer nanofibers, (2) being carbonized at 900 ^o^C. Reproduced with permission.^[^ ^89^ ^]^ Copyright 2020, Wiley‐VCH.

Heteroatom doping strategies have been studied for other electrocatalytic reactions, such as the oxygen reduction reaction and HER. Among them, fluorine doping is a promising strategy due to its strong electronegativity, inducing neighboring carbon atoms with a strong positive charge. Xie et al. synthesized a F‐doped carbon electrocatalyst with a high FE of 90% at a potential of 0.6 V_RHE_.^[^ ^85^ ^]^ DFT calculations reveal that the Gibbs free energy for *COOH adsorption on carbon atoms in pristine carbon exhibits the highest value. In contrast, the Gibbs free energy barrier for *COOH adsorption occurred at the fourth carbon atom (short for C4, the red atom in **Figure** **7**a) near the CF_2_ bonds and C1 (the blue atom in Figure 7a) is the lowest, resulting in enhanced catalytic performance. Although the FE was shown to be high in this work, the current density was only 0.24 mA cm^−2^. Recently, an F‐doped cagelike porous carbon (F‐CPC) was prepared by Ni et al.^[^ ^86^ ^]^ The F‐CPC electrocatalyst exhibited a FE of 83% for CO with a current density of 37.5 mA cm^−2^ at −1.0 V_RHE_. The author attributed the improved catalytic activity to a unique cagelike morphology, which possesses large surface area, enhanced local electrostatic field, and high local CO_2_ concentration near the surface of the F‐CPC (Figure 7b,c).

Moreover, it has been reported that pyridinic and graphitic N can act as catalytic sites for the highly efficient and stable conversion of CO_2_ to CO. N‐doped porous carbon (NC) electrocatalysts were prepared by using MOF precursors, which could tune the number of active N species by optimizing the calcination temperature and time.^[^ ^87^ ^]^ The obtained NC exhibits superior CO_2_RR performance with a high FE of 98.4% toward CO at −0.55 V_RHE_. Recently, Se, a nonmetallic chalcogen with a large atomic size, high polarizability, and rich d‐electrons, was used to prepare Se‐doped porous carbon nanosheets (Se‐CNs) electrocatalysts.^[^ ^88^ ^]^ The Se‐CNs electrocatalyst exhibited enhanced partial current density of CO (9 mA cm^−2^) compared with carbon nanosheets without Se doping and N‐ or F‐doped porous carbon at the same potential, indicating Se is a potential member of the metal‐free electrocatalyst family.

The tailoring of active centers of carbon‐based electrocatalysts for CO_2_RR by heteroatom co‐doping has been reported. Lin's group prepared an N, S co‐doped porous carbon membrane via electrospinning (Figure 7d).^[^ ^89^ ^]^ Since it is flexible and free‐standing, they could be directly utilized as an electrode, resulting in a high current density of −103 mA cm^−2^ at −0.7 V_RHE_. It can be readily extended to produce a wide range of heteroatom‐doped carbon membranes for high‐activity electrolysis and energy storage devices.

Beyond tuning the electronic structure of the electrocatalyst surface, functional group modification can also change the local microenvironment near the electrode surface. Thus, many researchers have focused on these strategies. An NH‐aziridine functionalized multi‐wall CNTs electrode showed a CO FE close to 90%.^[^ ^90^ ^]^ The exohedral functionalization strategy not only controls the chemical nature of the N‐dopant but also concentrates the N‐dopant at the nanomaterial surface (where the physical catalytic process occurs); thus, it offers a rational basis to understand the role of N‐sites and their neighboring carbons in the activation and conversion of small molecules. Oxygen‐containing groups on carbon materials are believed to induce high catalytic activity for some reactions. To prove this phenomenon, a series of metal‐free single‐layer graphene nanodisks with various oxygen‐containing groups were synthesized for CO_2_RR to generate formate.^[^ ^91^ ^]^ However, only the carboxyl groups were found to positively correlate with CO_2_RR catalytic performance. The enhanced catalytic activity originated from the synergistic effect between carboxyl groups and adjacent other types of groups (namely, hydroxyl, epoxide, and carbonyl) on graphene.

Compared with research on metal‐based catalysts, relevant investigations of carbon‐based electrocatalysts for CO_2_ reduction are relatively rare. More efforts should be devoted to promoting the development of highly efficient, carbon‐based electrocatalysts, both experimentally and computationally.

### 4.2 Metal‐Nitrogen Doped Carbon Catalysts

Metal‐nitrogen doped carbon materials (MNCs) containing non‐precious metals coordinated to earth‐abundant elements are currently one of the most promising candidates for CO_2_RR.^[^ ^92^ ^]^ MNCs combine the advantages of both homogeneous and heterogeneous catalysts.^[^ ^93^ ^]^ Usually, the metal sites in the SACs used for CO_2_RR are primarily transition metals (Fe, Ni), and CO is the primary reduction product (**Table** **6**). There are two main strategies to product MNCs, one is the use of high‐temperature pyrolyzed MNC catalysts, and the other is MOFs‐derived MNCs.

**Table 6:** Summary of CO_2_RR activity with recent reported M‐N‐C electrocatalyst

Sample	E [V_RHE_]	*j*_product_ [mA cm^−2^]	Main product	FE [%]	Ref.
Ni‐N‐C	−0.65	8.2	CO	96	^[^ ^94^ ^]^
Ni/Co‐N‐C	−0.9	51	CO	53	^[^ ^95^ ^]^
Ni‐N4@carbon	−0.83	90	CO	90	^[^ ^96^ ^]^
Ni‐N‐C	−1.0	200	CO	85	^[^ ^97^ ^]^
Co‐N‐C	−0.8	211	CO	92	^[^ ^98^ ^]^
Single Ni atom	−0.89	10.48	CO	71.9	^[^ ^101^ ^]^
Fe‐N‐C	−0.63	10	CO	80	^[^ ^102^ ^]^
Co‐N‐C	−0.68	18.1	CO	94	^[^ ^99^ ^]^

Pyrolyzing metal precursors with appropriate nitrogen and carbon sources is a facile approach to obtain a heterogeneous catalyst containing atomically dispersed metal sites. Theoretically, transition metals, such as Fe, Ni, Co, Mn, and Cr, can be used to produce MNCs catalysts. However, it has been found that Fe and Ni are more active than Co, Mn, and Cr in MNC for the reduction of CO_2_ to CO. Specifically, the main role of Fe is to reduce overpotentials, whereas Ni can drastically improve CO selectivity and reaction rates.^[^ ^94^ ^]^ Meanwhile, since Co‐N‐C is selective in producing H_2_, syngas evolution with controllable CO/H_2_ ratios can be achieved by tuning the ratio of Co and Ni.^[^ ^95^ ^]^ Recently, a model single‐atom Ni catalyst with a uniform structure and well‐defined Ni–N_4_ moiety on a conductive carbon support was designed to explore the active sites of MNCs catalysts in the electrochemical CO_2_RR.^[^ ^96^ ^]^ Operando XANE spectroscopy, Raman spectroscopy, and near‐ambient XPS results revealed that Ni^+^ in the Ni‐N‐C was highly active for CO_2_ activation and functioned as an authentic, catalytically active site for the CO_2_RR. However, the current densities of MNC catalysts are usually lower than those with a metal‐based electrode. To increase current density, a CO_2_ electrolyzer flow cell was constructed to provide CO partial current densities greater than 200 mA cm^−2^ and stable faradaic CO efficiencies around 85% for up to 20 h.^[^ ^97^ ^]^ Later, a flow cell with free‐standing Co‐N‐C electrodes was utilized to provide 211 mA cm^−2^ current density and 92% FE of CO.^[^ ^98^ ^]^

## 5 MOFs

MOFs have attracted tremendous attention due to their high surface area and tunable chemical structures. They consist of metal‐containing inorganic building blocks coordinated to multidentate organic ligands to form 3D interconnected networks. The ordered, porous heterogeneous network allows free permeation of electrolytes, counter ions, and dissolved CO_2_ into the interior of the crystal. Moreover, MOFs are typically composed of abundantly‐found elements, such as Fe, Co, Cu, Ni, and Mn, and organic ligands consisting of C, H, O, and N. The diversity of inorganic building blocks and organic ligands allows detailed and specific tailoring of MOF properties to optimize pore size and reactant diffusion and enhance affinity toward crucial intermediates.^[^ ^99^ ^]^ Generally, MOFs can be used as the precursor of electrocatalysts, the electrocatalyst and supports. MOF‐based electrocatalysts and their performances are listed in **Table** **7**.

**Table 7:** Summary of CO_2_RR activity with recent reported MOF‐based electrocatalyst

sample	E [V_RHE_]	*j*_product_ [mA cm^−2^]	Main product	FE [%]	Ref.
CR‐MOF	−1.2 [V_SHE_]	8	HCOOH	100	^[^ ^104^ ^]^
Zn‐ZIF	−1.1	12.8	CO	81	^[^ ^105^ ^]^
Ligand‐doped ZIF	−1.1	7	CO	90.57	^[^ ^106^ ^]^
Ag_2_O/layered ZIF	−1.2	25	CO	80.6	^[^ ^107^ ^]^

MOFs can be used as electrocatalytic precursors to prepare metal nanoparticles, single metal atom electrocatalysts, and heteroatoms‐doped carbon. Pyrolysis treatment can convert MOFs to carbon‐based electrocatalysts containing single metal atoms (SA). The highly exposed single metal sites demonstrate excellent mass activity toward CO_2_ reduction reactions, thus yielding high selectivity. For example, Wu's group prepared a series of atomically dispersed Co catalysts with different nitrogen coordination numbers at different pyrolysis temperatures (Co‐N_2_, Co‐N_3_, Co‐N_4_).^[^ ^100^ ^]^ The electrochemical results showed that the atomically dispersed Co with two‐coordinate nitrogen atoms (Co‐N_2_) achieved high activity and selectivity. Theoretical results prove that lower coordination numbers facilitate the activation of CO_2_ to the CO_2_^−^ intermediate. Meanwhile, Li et al. claimed that the edge‐hosted M−N_2+2_−C_8_ moieties bridging two adjacent armchair‐like graphitic layers are much more active than bulk‐hosted M−N_4_−C_10_ moieties embedded compactly in a graphitic layer for CO_2_RR.^[^ ^101^ ^]^ Although the optimal coordination number is still unclear, metal‐nitrogen sites are usually considered active species. However, there are a limited number of metal atoms in MOFs that can exclusively generate isolated metal‐nitrogen sites within carbon matrices after pyrolysis, so it is necessary to locate metal atoms on the catalyst surface to achieve high exposure of active sites. Solvent‐assisted metal or ligand exchange has been investigated for MOFs modification. For example, Li's group reported a single Ni atom electrocatalyst by ionic exchange between Zn nodes and adsorbed Ni ions.^[^ ^102^ ^]^ Moreover, Bao's group reported highly exposed Fe‐N active sites catalysts via ligand exchange treatment.^[^ ^103^ ^]^ High ligand exchange at the surface of MOFs can be achieved with steric effects; for instance, using bulky ligands to inhibit entry into the interior of MOFs. Besides microcosmic regulation, He's group proposed a strategy to maximize the accessible single‐atom cobalt sites via the construction of a free‐standing, cross‐linked, and high‐yield carbon membrane (denoted as CoSA/HCNFs).^[^ ^98^ ^]^ The improved single‐atom Co sites resulted in a high current density of 67 mA cm^−2^ in a typical H‐type cell and a current density of 211 mA cm^−2^ in a flow cell.

**Figure 8:**
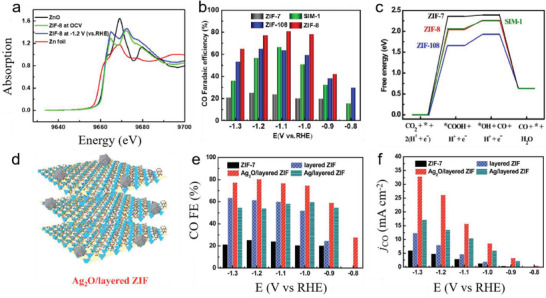
a) In situ XANES spectra of Zn K‐edge for ZIF‐8; b) The CO FE on ZIF‐8, ZIF‐108, ZIF‐7, and SIM‐1 at different applied potentials; c) DFT calculations of free energy diagrams with solvation effect corrections for CO_2_RR over Zn site (red) and ligand site (black) of ZIF‐8. Reproduced with permission.^[^ ^105^ ^]^ Copyright 2018, Elsevier. d) Schematic illustration of Ag_2_O/layered ZIF; The applied potential dependence of e) FEs and f) current densities for CO production over ZIF‐7, the layered ZIF, the Ag_2_O/layered ZIF and the Ag/layered ZIF. Reproduced with permission.^[^ ^107^ ^]^ Copyright 2017, Royal Society Chemistry.

The first application of MOF‐related catalysts for CO_2_RR was communicated in 2012 when a copper rubeanate MOF (CR‐MOF) was prepared by Hinogami et al. to electrochemically reduce CO_2_ into formic acid.^[^ ^104^ ^]^ Usually, metal centers are considered as active catalytic sites caused by diversified valence states. However, researchers found that the Zn center in ZIF‐8 was not reduced to metallic Zn during the electrochemical reduction process of CO_2_ (**Figure** **8**a).^[^ ^105^ ^]^ Meanwhile, MOFs with different ligands have distinct different electrocatalytic activities (Figure 8b,c), suggesting that active sites for CO_2_RR in ZIFs may be the ligands. Besides, it was found that ligands could enhance the electrocatalytic performance by creating unsaturated metal sites and improving charge transfer. For instance, Dou et al. boosted the activity of MOFs toward CO_2_RR via doping of the strong electron‐donating molecule of 1,10‐phenanthroline.^[^ ^106^ ^]^ Experimental and theoretical results revealed that the electron‐donating nature of phenanthroline enables charge transfer, which induces adjacent active sites at the sp_2_ C atoms in the imidazole ligand possessing more electrons and facilitates the generation of *COOH, resulting in improved activity and FE toward CO production.

Extensive research efforts have been invested in applying MOFs as supports as they can benefit mass transport during the electrochemical CO_2_RR. Bao's group reported the construction of a Ag_2_O/layered ZIF composite structure by mixing pre‐synthesized layered ZIF‐7 with AgNO_3_ aqueous solution, followed by refluxing at 100 °C (Figure 8d).^[^ ^107^ ^]^ The Ag_2_O/layered ZIF electrode showed much higher CO FE (≈80%) (Figure 8e). Importantly, it showed a high current density of CO (≈32 mA cm^−2^) (Figure 8f). The improved performance could be attributed to the facilitated mass transport by the high specific surface area of the Ag_2_O/layered ZIF and synergistic effects between Ag_2_O NPs and layered ZIF.

Even though the FE and current density of the MOF‐derived electrocatalyst have been improved by various strategies, the main product of MOF and MOF‐derived electrocatalyst is usually C_1_ product. Considering the flexibility of MOF composition, it is promising to develop a MOF‐based electrocatalyst to produce multicarbon products.

## 6 Conclusions

In summary, the recent progress of electrocatalysts for CO_2_RR has been exemplified. Although an increasing number of electrocatalysts for electrochemical CO_2_ reduction reactions has been explored in recent years, there are still some issues that must be more deeply investigated, such as the catalytic mechanism, specific adsorption/desorption, and intrinsic activity of materials. To design more efficient electrocatalysts with high activity and selectivity, suggestions for novel CO_2_RR electrocatalysts include the following:

1. An electrocatalyst with high product selectivity. The product selectivity of existing electrocatalytic CO_2_ reduction systems is still low. For the generation of two‐electron products, such as formate and CO, the reported FE is high at one given potential. However, the overpotential is still quite high compared with the theoretical value. For the selective production of multi‐carbons, the reported electrocatalyst presents low FE. There is still a lack of systematic understanding of the reaction mechanism, and it remains a significant challenge in determining the critical factor or factors that enhance the selectivity of one particular product over another with a high FE. Furthermore, a high current density and a broad potential window are required in industry.
2. In situ techniques with high surface sensitivity. Generally, there are a few common baseline inquiries for the CO_2_RR: first, the reaction environment effect, such as cation, pH value, concentration, and solvent; second, the critical intermediates, such as the type and configuration, for various reaction pathways and selectivities on each electrode; third, catalyst evolution, such as the nanostructure and active sites under the reaction conditions. To address these issues, more precise and surface selective in situ techniques must be developed. For example, TEM is a powerful tool to record atomic and morphological changes, and the liquid phase TEM has already been used in studying OER electrocatalysts. Thus, it may be used to identify catalytically active surfaces during the CO_2_RR process. Meanwhile, in situ sum‐frequency generation, as a second‐order nonlinear optical technology with interface selectivity and sensitivity, can be used to investigate the molecular structure at the interface without influence from the electrode, making it one of the most effective means for interface research. It can be used to investigate chemical bond changes at various applied potentials. Finally, characterization techniques should not only be quasi in situ, but flow‐cells should also be considered as a mode to enhance experimental, real time data.
3. A deeper understanding of the reaction mechanism for CO_2_RR. To understand the pathways during CO_2_ reduction, DFT calculations have been employed to explain the high selectivity of certain products. Based on the development of nanostructured materials and alloys, more theoretical calculations have been carried out based on different models. This has been helpful as the reaction pathways to different products can be quite complicated. Thus, mechanistic understanding is still not systematic. A deeper understanding of the reaction mechanisms should be helpful for the rational design of novel catalysts. Fortunately, combining deeper mechanistic understanding with the development of in situ/operando characterization techniques should provide in‐depth insights.
4. More feasible preparation methods. It is positive to read the reports of nanostructured electrocatalysts with high FE for CO_2_ conversion. However, most of the studies focused on catalyst optimization without considering costs. The tedious preparation routes and vast usage of expensive precursors and surfactants usually bring high costs. Moreover, few studies reported catalyst preparation on a large or industrial scale. To move from academic studies to industrial applications, decreasing the cost and increasing the production of these nanocatalysts while maintaining the superior properties of nanostructured catalysts is a great challenge. Therefore, simplifying the production process and large‐scale production of working electrodes could significantly improve the path toward industrialization.

In brief, future trends in the field of electrochemical CO_2_ reduction should focus on the preparation of nanostructured catalysts with high selectivity, activity, and stability. Although recent advancements in the development of solid‐state catalysts for electrochemical conversion of CO_2_ are still far from large‐scale applications, they offer valuable information and background for catalyst optimization to meet the required activities for industry. With the help of in situ characterization techniques and theoretical calculations, we believe a deeper fundamental understanding of the reaction mechanisms can be achieved. From this knowledge, we believe practical information can be gathered for the rational design of novel electrocatalysts. Furthermore, the pathway from academic studies to industrial production and commercialization is expected to be realized in the future.

## Conflict of Interest

The authors declare no conflict of interest.
